# Multidimensional Analysis of Major Depression: Association Between BDNF Methylation, Psychosocial and Cognitive Domains

**DOI:** 10.3389/fpsyt.2021.768680

**Published:** 2021-12-14

**Authors:** María Marcela Velásquez, Yvonne Gómez-Maquet, Eugenio Ferro, Wilmer Cárdenas, Silvia González-Nieves, María Claudia Lattig

**Affiliations:** ^1^Centro de Investigaciones Genéticas en Enfermedades Humanas, Departamento de Ciencias Biológicas, Universidad de los Andes, Bogotá, Colombia; ^2^Departamento de Psicología, Universidad de los Andes, Bogotá, Colombia; ^3^Instituto Colombiano del Sistema Nervioso, Clínica Montserrat, Bogotá, Colombia; ^4^SIGEN alianza Universidad de los Andes – Fundación Santa Fe de Bogotá, Bogotá, Colombia

**Keywords:** major depressive disorder, BDNF, maladaptive cognitive schemas, methylation, epigenetics, family history of depression

## Abstract

Major Depression is a complex disorder with a growing incidence worldwide and multiple variables have been associated with its etiology. Nonetheless, its diagnosis is continually changing and the need to understand it from a multidimensional perspective is clear. The purpose of this study was to identify risk factors for depression in a case-control study with 100 depressive inpatients and 87 healthy controls. A multivariate logistic regression analysis was performed including psychosocial factors, cognitive maladaptive schema domains, and specific epigenetic marks (BDNF methylation levels at five CpG sites in promoter IV). A family history of depression, the cognitive schemas of impaired autonomy/performance, impaired limits, other-directedness, and the methylation level of a specific CpG site were identified as predictors. Interestingly, we found a mediating effect of those cognitive schemas in the relationship between childhood maltreatment and depression. Also, we found that depressive patients exhibited hypomethylation in a CpG site of BDNF promoter IV, which adds to the current discussion about the role of methylation in depression. We highlight that determining the methylation of a specific region of a single gene offers the possibility of accessing a highly informative an easily measurable variable, which represents benefits for diagnosis. Following complete replication and validation on larger samples, models like ours could be applicable as additional diagnostic tools in the clinical context.

## Introduction

Major Depressive Disorder (MDD) is a highly prevalent and vastly complex clinical condition, which requires a multidimensional approach in its study (1–4). Several studies have highlighted that the risk of suffering from depression is related to cognitive patterns acquired during childhood, shaping the individual's ability to cope with daily life events during adulthood (5–9). In this regard, early environmental conditions, including maltreatment and a family history of depression, have been linked to the disorder (10, 11). There is also evidence of the mediating role of cognitive factors in the relationship between childhood maltreatment and subsequent adult psychopathology, including higher risk of developing depression (12–14).

Negative affective biases related to early life adversity (ELA) can increase the risk of developing MDD, which is known as the cognitive diathesis-stress model of depression (15). This cognitive model suggests that the negative attributional processes that the individual activates against life events are associated with the development of depressive symptomatology (16–18). Besides, the theory of early maladaptive schema (EMS) domains emphasizes the role of the cognitive dimension in the developing psychopathology, asserting that some patterns and tendencies acquired during childhood can represent an additional risk of suffering some kind of mental disorder (19, 20). EMS has been defined as pervasive and dysfunctional beliefs about the self and the relationship with others, which are classified into five domains: “Disconnection and Rejection” (DR), “Impaired Autonomy and Performance” (IAP), “Impaired Limits” (IL), “Other-directedness” (OD), and “Overvigilance and Inhibition” (OIN) (19, 21). There is a well-established link between developing EMS and the impossibility of satisfying psychological needs, such as manifesting autonomy or secure attachment, which is also related to parental care (12). For this point, there is evidence suggesting that individuals whose families have a history of depression are more likely to develop the disorder, given that they are exposed to adverse conditions created in the familiar context (22).

Biological mechanisms—epigenetics, for example—have been identified as crucial mediators of psychosocial factors and subsequent emerging risk for mental illness (23). The analysis of epigenetic marks associated with depression seems to be a promising path for the study of MDD. However, not all studies have shown differences in the methylation levels between depressive patients and controls (24–26), and there have been conflicting results, with either increased or decreased methylation within the promoter regions and its role in the pathogenesis of affective disorders (27–29). The methylation level within the brain-derived neurotrophic factor (BDNF, Gene ID: 627) has been one of the most studied epigenetic marks since this gene codes for a neurotrophin involved in the development and functioning of the nervous system, especially in neuronal growth, proliferation, and survival (25, 30, 31). The BDNF exon IV promoter region has been of particular interest as it is critical for activity-dependent transcription and includes several CpG sites liable to methylate (26, 27, 32). Despite these contrasting findings when reporting BDNF methylation in MDD, what seems clear is that ELA, including childhood maltreatment and neglect, is a key predictor for major depressive disorder, as it has been found to epigenetically affect critical behavioral systems (33).

The multidimensional nature of the disorder, and its wide phenotype spectrum has supposed a challenge for diagnosis. It has been reported that general practitioners correctly identify depression in about half of the cases. The rate of accurate diagnosis through clinical measurements has a wide variation between countries, with over-detection (false positives) as a latent problem (34), a situation that increase the need to further elucidate the nature of the disorder. Thus, this study intended to determine which psychosocial and cognitive variables (including early life adversity and maladaptive schema domains) and epigenetic marks (BDNF methylation levels at five CpG sites in promoter IV) could be useful to establish a multivariate model to differentiate individuals diagnosed with MDD from healthy controls.

## Methods

### Study Design and Subjects

A case-control study was performed to identify associations between Major Depressive Disorder and psychosocial (childhood adversity, early maladaptive schema domains and family history of depression), epigenetic (BDNF methylation levels at five CpG sites in promoter IV), and socio-demographic variables. Inpatients were recruited from two psychiatric hospitals in Bogotá. All of them had a primary diagnosis of MDD according to the ICD-10 criteria (35), and confirmed by the MINI structured interview for DSM-IV (36). Other inclusion criteria for the cases were that they had to be over 18 years of age and have at least completed elementary school (to guarantee the understanding of the measuring instruments). Exclusion criteria included: bipolar depression, comorbidity with substance abuse or dependence, psychotic disorders, dementia, and/or delirium. The control group was made up of healthy participants, recruited via a screening procedure from the general population. The following inclusion criteria were considered for the controls: individuals without past or present MDD diagnosis, subjects aged least 18, with completed elementary school. Exclusion criteria for the control group included the diagnosis of any psychiatric condition (addiction, bipolar disorder, organic mental disorder, psychotic disorder) and family relationship with a case subject. The collection of the sample was non-probabilistic for convenience. The participant flow diagram is presented in the Supplementary Figure 1.

### Psychological Measures

Each participant completed the following standardized questionnaires.

A personal questionnaire consisting in a clinical research interview designed to assess family and personal risk factors, including information about physical or psychological early maltreatment or neglect, as well as any family history of depression.

A Mini-International Neuropsychiatric Interview (36), which is a structured diagnostic interview considered a gold standard to confirm inclusion criteria for all eligible patients and rule out mental disorders from controls.

A Schema Questionnaire-Short Form (YSQ-SF) (37), which assesses the maladaptive schemas proposed by Young (38) and was used to evaluate the presence of cognitive maladaptive schemas in the participants. Participants graded each of the 75 items of the test, providing a score between 1 (does not fit) and 6 (perfect fit), with higher scores indicating greater maladaptive schemas (Cronbach's alpha 0.82).

### Molecular Analysis

After psychological measures were completed, a blood sample was obtained from each individual to perform molecular analysis. The DNA was isolated from leukocytes with the DNA 2,000 kit (Corpogen) and its concentration was determined with a NanoDrop (Thermo Scientific) spectrophotometer. Four hundred nanograms of the DNA were used for bisulfite conversion according to the manufacturer's protocol (EZ DNA Methylation Gold Kit; Zymo Research, CA, USA). The PCR reactions were carried out in a total volume of 20 μl using 1 μl mmol of each primer, 10 μl of GoTaq Hot Start Master Mix (Promega, USA), and 10 μl of nucleotides-free water. We used primers to amplify the region of interest, independently of the methylation status: 5′- TGATTTTGGTAATTNGTGTATT−3′ and 5′- CTCCTTCTATTCTACAACAAAAAA−3′. The amplification protocol involved a denaturation cycle (5 min, 95°C), 45 cycles of denaturation (1 min, 95°C), annealing (45 s, 57°C), and extension (1 min, 72°C), followed by a final extension cycle (5 min, 72°C) terminating at 4°C. PCR products were separated onto 2% agarose gel to verify the amplification of the region of interest.

**Figure 1:**
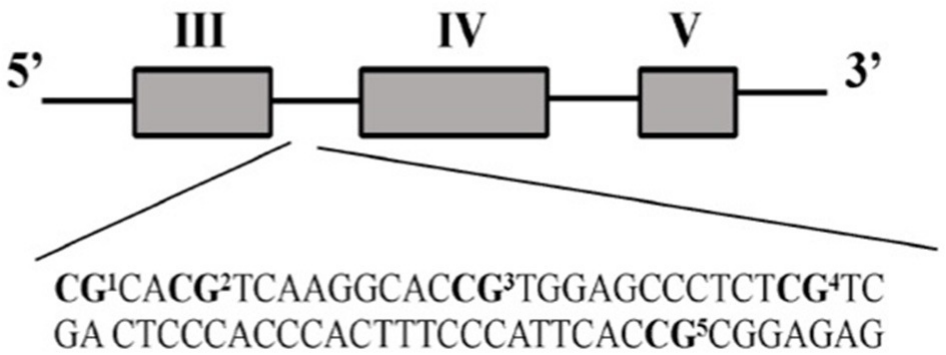
BDNF promoter IV region evaluated in this study (chr11: 27,701,519-27,701,826 UCSC Genome Browser Human GRCh38/hg38 Assembly).

The five CpG sites targeted in our analysis encompass a 66 bp BDNF promoter IV region (Figure 1). We assessed the methylation levels of the CpG positions through direct sequencing of bisulfite-treated DNA (BSP).

The amplified fragment was sequenced by Sanger's method at the university sequencing center. DNA methylation percentages were measured using ESME (Epigenetic Sequencing Methylation Software) (39), which performs a quantitative estimate with a specific algorithm that normalizes the signal of the sequencing, corrects incomplete conversion problems, and neutralizes possible artifacts in the nucleotide signals.

Population substructure analysis was assessed by 46 ancestry informative markers (AIMs) according to the protocol described by Pereira et al. (40). The PCR products were prepared for posterior capillary electrophoresis (41) and then analyzed for genotyping with GeneMapper (42). The differences in the ancestry proportions between patients and controls were estimated using STRUCTURE (43).

### Statistical Analysis

Statistical analysis was conducted using RStudio (44). A significance level of 5% was used for all analyses. Fisher's exact tests and *T*-test were used to determine group differences for sociodemographic variables. Univariate logistic regression analyses (including age and gender as covariates) were first performed for the psychosocial, cognitive, and epigenetic features to identify associations with MDD. Next, a multivariate logistic regression was conducted including the features with a *p* value of < 0.05 in the univariate analysis. This model also included age and gender as covariates. The factors with a *p* value of 0.05 or less in the multivariate analysis were identified to be significantly correlated with MDD. Variance Inflation Factor (VIF) was used to determine the multicollinearity of the data within the model. A causal mediation analysis was carried out to test the hypothesis that the cognitive domains significantly associated with MDD act as mediating factors between childhood maltreatment and the probability of developing depression. Considering the limited sample-set size, a 10-fold cross validation strategy with 100-round classifications was used to evaluate the performance of the model. Training was performed using 80% of the total data set and testing was performed with the remaining 20% (45).

### Ethics

The study protocol and informed consent were approved by the Institutional Ethics Committee of each institution. Prior to any procedure, written informed consent was obtained from all participants, in agreement with the Declaration of Helsinki.

## Results

### Participant Characteristics

A total of 100 inpatients clinically diagnosed with major depression and 87 healthy controls were recruited for the study. Demographic characteristics and ancestry information of the participants are presented in Table 1. Our sample was initially paired by gender. However, after removing some control samples that did not meet the quality criteria for the methylation analysis, this match was lost.

**Table 1:** Demographic characteristics and population substructure of participants.

**Variable**	**Depression group (*N* = 100)**	**Control group**	***p* value**
**(*****N*** **=** **87)**	***p***		
Sex (female), *N* (%)	64 (64.0)	69 (79.3)	0.02^*^
Age (years), mean ± SD	34.28 ± 13.27	35.59 ± 9.20	0.43
History of marriage, *N* (%)	49 (49.0)	47 (54.02)	0.55
History of divorce, *N* (%)	6 (6.0)	10 (11.49)	0.20
Presence of spouse, *N* (%)	43 (43.0)	37 (42.52)	1.0
**Ancestry**
African (%), mean ± SD	11.70 ± 0.06	11.67 ± 0.07	0.97
European (%), mean ± SD	50.83 ± 0.18	51.15 ± 0.16	0.90
Native-American (%), mean ± SD	37.45 ± 0.16	37.17 ± 0.17	0.91

* *p < 0.05*.

*Fisher's exact test for count data; T test for continuous variables*.

### Univariate Analyses

Univariate logistic regression analyses showed that having a family history of depression, the exposure to childhood adversity, higher scores for cognitive schemas, as well as the methylation level of three CpG sites are significantly associated with depression (Supplementary Table 1).

### Risk Factors for Depression

The features that showed a *p* value of < 0.05 in the univariate analysis were included in the multivariate logistic regression model. This analysis was adjusted by age and gender and yielded a model according to which a family history of depression, the early maladaptive schema domains of impaired autonomy/performance, impaired limits and other-directedness showed a strong association to MDD. The schema domain of disconnection/rejection almost reached statistical significance (*p* = 0.054). Regarding methylation, the multivariate analysis revealed that only one CpG site (BDNF IV CpG3: Chr11: 27723204-CRCh37/hg19) was related to the disorder (Table 2).

**Table 2:** Results of the multivariate logistic regression analysis.

**Variable**	***p***	**Odds ratio**	**95% CI**
Sex	0.67	0.69	0.12–3.91
Age	0.16	1.04	0.98–1.10
FHDEP	0.002^**^	12.68	2.81–78.3
C. Adversity	0.20	2.45	0.63–10.07
IAP-Domain	0.005^**^	1.49	1.14–2.02
DR-Domain	0.054	1.26	1.00–1.63
OIN-Domain	0.333	0.89	0.71–1.11
IL-Domain	0.020^*^	1.21	1.03–1.46
OD-Domain	0.045^*^	1.18	1.00–1.40
CpG1	0.56	0.98	0.91–1.04
CpG3	0.008^**^	0.91	0.85–0.97
CpG4	0.27	1.06	0.94– 1.21

*FHDEP, Family History of Depression; C. Adversity, Childhood Adversity; IAP-Domain, Impaired Autonomy and Performance Schema Domain; DR-Domain, Disconnection and rejection Schema Domain; IL-Domain, Impaired limits Schema Domain; OD-Domain, Other-directedness Schema Domain; OIN-Domain, Overvigilance/inhibition Schema Domain*.

** *p < 0.01;*

* *p < 0.05*.

Considering the five variables that are significantly associated with MDD, we formulated the following logistic regression equation: logit (D) = ln (D/1-D) = −12.37 + 2.54X_1_ + 0.40X_2_ + 0.19X_3_ + 0.16X_4_ + (−0.08X_5_) (D: probability of predicting MDD [0–1], X_1_: family history of depression [yes, no], X_2_: IAP-Domain [continuous score], X_3_: IL-Domain [continuous score], X_4_: OD-Domain [continuous score], X_5_: Methylation level at CpG3 [percentage]. Of the five factors, only the methylation level at CpG3 exhibited a negative association with the disorder, this being hypomethylated in patients compared to controls.

Tests to determine whether the data met the assumption of collinearity indicated that multicollinearity was not a concern in the resulting model (VIF <5). The 10-fold cross validation analysis showed that our model has an accuracy of 86% (95% CI: 0.71–0.95, *p* < 0.001), a sensitivity (true positive rate) of 90% and a specificity (true negative rate) of 81% (Kappa = 0.72).

### Moderation Analysis

**Figure 2:**
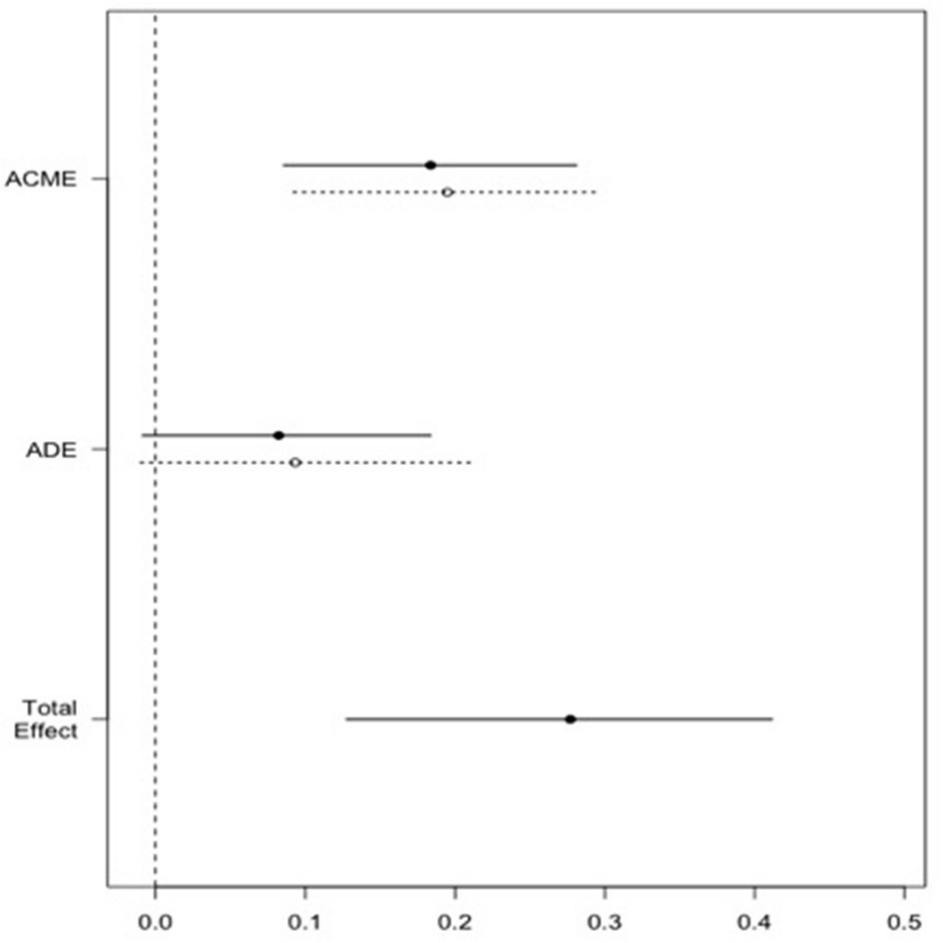
Effect of childhood maltreatment on likelihood of MDD through cognitive schemas. Causal mediation analysis results. ACME stands for average causal mediation effects of childhood maltreatment on the likelihood of MDD through the cognitive schemas; ADE stands for average direct effects of childhood maltreatment on the likelihood of MDD; Total effect stands for the total effects (direct and indirect) of childhood maltreatment on the likelihood of MDD.

The causal mediation analysis showed that the additive score of the cognitive schemas of impaired autonomy/performance, impaired limits and other-directedness acts as a mediating variable between childhood maltreatment and the probability of being diagnosed with major depression (Prop. Mediated = 68%, 95% CI: 0.09–0.29, *p* < 0.0001). The estimated average of the mediating effect is represented in Figure 2.

## Discussion

A multifactorial logistic model was evaluated in the present study, including psychosocial, cognitive, and epigenetic factors associated with MDD. Regarding the family history of depression, recent evidence reports that familial risk increases the probability of individual lifetime depression (22, 46). Although it has been argued that the transgenerational effect of family risk may have a genetic background (47), the disorder's low heritability has encouraged the exploration of other hypotheses. It seems that familial risk has a strong link with early adverse conditions since high-risk families are more likely to experience a more significant number of challenging situations, which predicts the development of more severe depression (48). Concerning early adversity, there is a large body of evidence that experiencing abuse or maltreatment during childhood accounts as a risk factor in the development of psychopathology during adulthood (11, 49). The multivariate logistic regression in this study did not reveal a significant association between childhood maltreatment and MDD. This is explained by the mediating effect that we found of the cognitive schemas in the relationship of childhood adversity and the likelihood of depression. This finding is consistent across different studies confirming that cognitive factors could act as mediators between early maltreatment and the subsequent risk of developing psychopathologies (13, 14). This is comprehensible since, according to schema therapy (19), neglected children are at risk of developing EMS. Besides, some authors have pointed out that childhood adversity can indirectly predict depression through cognitive vulnerabilities, including dysfunctional schemas (50).

Additional evidence supporting the previous hypothesis states that the schema domains of impaired autonomy/performance and disconnection/rejection mediate the relationship between childhood maltreatment and depression (51). It is also consistent with prior research establishing that maladaptive schemas of impaired autonomy/performance, impaired limits and other-directedness have a strong association with MDD (5, 52–55). Schemas represent a cognitive dimension, which seems highly stable over time in depressed patients (20). Impaired autonomy and performance are related to the inability to cope in everyday life, which affects the individual in many dimensions, involving dependence and an underdeveloped self (21). Not surprisingly, it has been reported that the reduction of depressive symptoms in patients under treatment was strongly associated with a reduction in the punctuation of this schema (56). Impaired limits involve difficulties in setting internal limits, assuming responsibility or even setting long-term goals (57) and it have been identified as a predictor of depression severity (58). The other-directedness EMS implies subjugation, self-sacrifice and constant seeking for recognition, as well as a tendency to respect other's desires at the expense of one's own needs (57). This domain has also been highlighted for its relation with depressive symptoms (59) as well as for its mediating role between co-rumination and depression (60).

One of the biological processes underlying the etiology of major depression is epigenetics, which comprises molecular mechanisms, like DNA methylation, that modulate gene expression in response to extrinsic or intrinsic signals (61). In this study, we evaluated the methylation level at specific CpG sites in BDNF promoter IV. The brain-derived neurotrophic factor is a neurotrophin that has been widely associated with major depressive disorder since abnormalities in its expression produce dysfunction in circuits that compromise emotional and cognitive functions (24, 62, 63). Our association results for the CpG3 site in BDNF promoter IV suggest that individuals with elevated methylation levels are less likely to show a depressive phenotype. This finding contrasts with prior research establishing that individuals with depression have lower levels of BDNF than healthy controls (64, 65) which, hypothetically, would be related to higher levels of methylation in patients. However, results concerning the methylation of BDNF and its role in depression have been ambiguous. There is evidence showing higher methylation in some CpG regions of BDNF promoters I and IV of depressed patients than healthy controls (64), highlighting childhood adversity as a mediating factor of these differences (65). In contrast, other authors report that those regions can appear as either hypomethylated or hypermethylated in clinical populations (66). A study involving mothers and their newborns revealed that prenatal depressive symptoms predicted decreased BDNF IV DNA methylation in infants (28). Specifically, they found hypomethylation at the same CpG site assessed in the present study in newborns who were prenatally exposed to maternal stress compared to controls.

The differences found within that specific CpG site could imply changes in BDNF mRNA and protein expression since it is adjacent to the binding site for the transcription factor CREB (cAMP response element-binding), which modulates gene transcription via a DNA methylation-dependent mechanism (67, 68). Another study demonstrates that healthy individuals with a family history of depression exhibited higher peripheral BDNF levels (69), presumably suggesting hypomethylation in those individuals with higher familial risk. There is also evidence of DNA hypomethylation in fragments of BDNF IV in patients who have schizophrenia compared to controls (70).

It is important to note that CpG3 includes binding sites for the glucocorticoid receptor (GR), as shown on the PROMO platform (71). This gene is one of the most important for regulating the stress response through the Hypothalamic-Pituitary-Adrenal (HPA) axis and modulation of peripheral cortisol levels. Concerning depression, it has been shown that there is an alteration in the sensitivity of the GR, which causes control failure in the cortisol levels after activation of the HPA axis (72). Likewise, certain antidepressants directly affect glucocorticoid receptors, increasing their functionality and expression (73).

Our model underscores the importance of the individual's cognitive dimension and the echoes that it can produce at specific epigenetic marks to understand the depressive disorder better. Accordingly, to consider a complex disorder like major depression, we need a powerful kaleidoscope of factors that can help us to understand fractions and to gain a general perspective of a condition that involves many biological systems and, therefore, affects the individual in multiple dimensions in their daily life. Altogether, the results of our study suggest that a model including psychosocial, cognitive, and epigenetic factors could help differentiate depressive patients from healthy controls and, therefore, could contribute to clinical diagnosis. Indeed, it has been highlighted that biological and cognitive measurements will be crucial, beyond traditional symptom-based diagnosis, to subtyping and redefining psychiatric disorders (3, 74). Additionally, the inclusion of a biological variable, like DNA methylation of a critical gene previously related to depression, is an essential step toward future strategies for treatment and prevention. In the future, it would be benefit to explore association with other approaches, such as the endophenotypes described for depression and anxiety (75), including variables related to Behavioral Activation System (BAS) and Behavioral Inhibition System (BIS), which can act as moderators between depressive symptoms and live events (76). Also, adding functional analysis to clarify the effect of the methylation level at BDNF promoter IV, as well as testing the proposed model in samples from different populations could be very useful in future studies.

## Data Availability Statement

The data that support the findings of this study are available from the corresponding author upon request.

## Ethics Statement

The studies involving human participants were reviewed and approved by Ethics Committees of the University of the Andes and the Montserrat clinic. The patients/participants provided their written informed consent to participate in this study.

## Author Contributions

MV, YG-M, EF, and ML: conceived and designed the research. MV, YG-M, EF, and WC: collected the data. MV, YG-M, SG-N, and WC: performed the analysis. MV, YG-M, EF, SG-N, and ML: wrote the paper. All authors contributed to the article and approved the submitted version.

## Funding

This study was financed by the faculty of sciences, the vice-rectorate research of the University of los Andes and by a research grant (712.2015/908/120471250970) from Minciencias. They provided resources, but did not actively participate in any stage of the research.

## Conflict of Interest

The authors declare that the research was conducted in the absence of any commercial or financial relationships that could be construed as a potential conflict of interest.

## Publisher's Note

All claims expressed in this article are solely those of the authors and do not necessarily represent those of their affiliated organizations, or those of the publisher, the editors and the reviewers. Any product that may be evaluated in this article, or claim that may be made by its manufacturer, is not guaranteed or endorsed by the publisher.
