# Supplementary material for: Multidimensional Analysis of Major Depression: Association Between BDNF Methylation, Psychosocial and Cognitive Domains
Source: Front Psychiatry. 2021 Dec 14;12:768680. doi: 10.3389/fpsyt.2021.768680 (PMC8712447; doi:10.3389/fpsyt.2021.768680)
Supplement: Supplementary file 1 [file Table_1.DOCX]

**Supplementary Table 1.** Results of univariate logistic regression analyses of psychosocial and epigenetic variables

| **Variable** | **p value** | **Odds Ratio** | **95% CI** |
| --- | --- | --- | --- |
| FHDEP | < 0.001^***^ | 5.60 | 2.81 – 11.69 |
| C. Adversity | < 0.001^***^ | 3.83 | 2.01 – 7.56 |
| IAP-Domain | < 0.001^***^ | 1.77 | 1.53 – 2.13 |
| DR-Domain | < 0.001^***^ | 1.54 | 1.39 – 1.75 |
| IL-Domain | < 0.001^***^ | 1.42 | 1.29 – 1.58 |
| OD-Domain | < 0.001^***^ | 1.48 | 1.33 – 1.67 |
| OIN-Domain | < 0.001^***^ | 1.35 | 1.24 – 1.50 |
| CpG1 | 0.03^*^ | 0.96 | 0.93 – 0.99 |
| CpG2 | 0.11 | 1.04 | 0.99 – 1.11 |
| CpG3 | 0.001^**^ | 0.95 | 0.92 – 0.98 |
| CpG4 | 0.038^*^ | 1.06 | 1.00 – 1.14 |
| CpG5 | 0.062 | 1.05 | 1.00 – 1.12 |

FHDEP: Family History of Depression; C. Adversity: Childhood Adversity; IAP-Domain: Impaired Autonomy and Performance Schema Domain; DR-Domain: Disconnection and rejection Schema Domain; IL-Domain: Impaired limits Schema Domain; OD-Domain: Other-directedness Schema Domain; OIN-Domain: Overvigilance/inhibition Schema Domain. *** p< 0.001; ** p< 0.01; * p< 0.05.
