# Supplementary figures and images for: Multidimensional Analysis of Major Depression: Association Between BDNF Methylation, Psychosocial and Cognitive Domains
Source: Front Psychiatry. 2021 Dec 14;12:768680. doi: 10.3389/fpsyt.2021.768680 (PMC8712447; doi:10.3389/fpsyt.2021.768680)

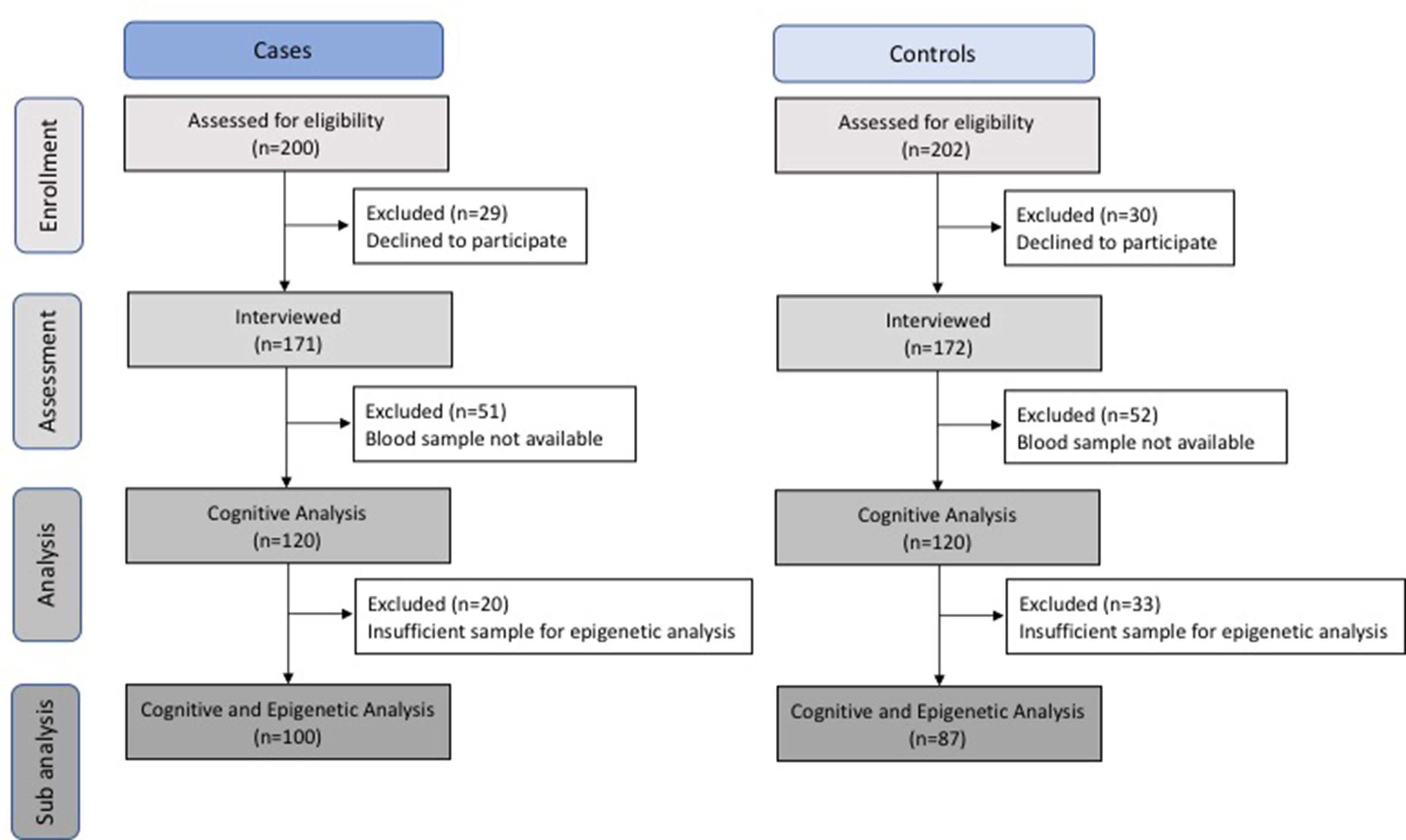

Supplement: Supplementary file 2 [file Image_1.jpg]
